# The Internal Secretions and the Principles of Medicine

**Published:** 1908-06

**Authors:** 


**The Internal Secretions and the Principles of Medicine.** By CHARLES E. DE M. SAJOUS, M.D. Vol. II. Pp. xxv., 801–1873. Philadelphia: F. A. Davis Company. 1907.

In 1905 the late Sir Michael Foster referred to our knowledge of metabolism as consisting mostly of gaps and guesses, and Dr. Sajous has, in his work on the internal secretions, made a bold attempt to bridge over these gaps. The structure which he has put together is composed chiefly of the theory that the secretion of the suprarenal glands is the dynamic element in all the vital processes of the organism, and the keystone of his bridge is the hypothesis that the pituitary body acts as a control centre, presiding over the activity of the suprarenals, and in addition influencing and co-ordinating the movements of muscles, including the striped and unstriped varieties, also that the pituitary body receives all sensory impulses.

In Chapter XVI. the properties of the pituitary are treated on at great length and in a methodical manner, experimental and pathological data being produced to prove the importance of this organ as a nerve centre, as a governing centre to the sympathetic system, as a centre of common sensibility, and as a general motor centre.

The experiments of Marinesco, Vassali and Sacchi are quoted, in which the pituitary was destroyed by cautery. All the animals died when the removal had been complete, but before death they showed diminution of body temperature, anorexia and lassitude, muscular twitchings and tremors, developing into spasms and dyspnœa.

In support of the statement that the posterior tube of the pituitary is the seat of the sympathetic centre, reference is made to the experiments of Cyon, Masay and Brown-Séquard, the evidence showing that even slight pressure upon this organ caused variation in the blood pressure and a marked reduction of heart frequency. The observations of Cyon were confirmed recently by Masay, who showed that mechanical or electrical excitation of the pituitary caused a rise in the blood pressure from 81 to 200 mm. of Hg.

In support of the statement that the pituitary is the receiver of sensory impressions, the case of Goltz's dog is cited among others. In this instance the dog, after being deprived of its cerebral hemispheres, reacted promptly to tickle sensations, limped when hurt, promptly raised its feet when these were placed in cold water, etc.

And that the removal of the pituitary abolishes sensation was shown by Cyon, who observed that its removal annulled nasal sensory phenomena such as sneezing, etc., and that all the nerves, including the fifth and the glosso-pharyngeal, lost their reflex influence.

Again, clinical evidence is deduced from such cases as acromegaly and sarcoma in which the posterior lobe is involved alone or in conjunction with the anterior lobe, as it is found that these conditions can provoke a great variety of motor disorders, such as spastic gait or paresis, disturbance of speech, paralysis of ocular muscles, muscular asthenia, unsteadiness of gait and impairment of co-ordination.

A large amount of space is devoted to applied therapeutics as regarded in the light of the author's views, and the subject has been dealt with very fully.

